# The long-term studies of osmotic membrane distillation

**DOI:** 10.1007/s11696-017-0261-1

**Published:** 2017-08-03

**Authors:** Marek Gryta

**Affiliations:** Faculty of Chemical Technology and Engineering, West Pomeranian University of Technology, Szczecin, ul. Pułaskiego 10, 70-322 Szczecin, Poland

**Keywords:** Osmotic distillation, Fouling, Scaling, Hydrophobic membrane

## Abstract

The results of osmotic membrane distillation carried out for 2.5 years were presented in this work. The influence of the process conditions, such as temperature and brine concentration on the permeate flux, was investigated. The saturated NaCl solutions and distilled water were used as a stripping solution and feed, respectively. A continuous regeneration of stripping solution was conducted using a method of natural evaporation from the surface of Białecki rings to the air surrounding the installation. The possibilities of application of Accurel PP S6/2 hydrophobic polypropylene membranes were tested. It was studied whether a saturation stripping solution does not cause scaling and wettability of membranes. It was found that most of the pores in the used membranes were non-wetted, and the salt retention over 99% was maintained during a study period. However, the obtained permeate flux was decreased by 10–20%. The SEM examinations revealed that it was caused by amorphous deposit, which was formed on the membrane surface on the brine side. The SEM–EDS analysis demonstrated that the deposit composition mainly included Si and O.

**Electronic supplementary material:**

The online version of this article (doi:10.1007/s11696-017-0261-1) contains supplementary material, which is available to authorized users.

## Introduction

The osmotic membrane distillation (OMD) is a low-temperature method for solution concentration by water evaporation. In this process both sides of a porous hydrophobic membrane are in contact with two aqueous solutions (feed and striping solution—usually brine) (Bui and Nguyen 2005; Vargas-Garcia et al. 2011). The hydrophobic property of the used membrane prevents a penetration of separated solutions into the membrane pores and the pores are filled only by the gas phase (Goh et al. 2013; Zambra et al. 2015). The presence of dry pores enables the transfer of water vapor from the feed to brine. Usually, the membranes made from polyvinylidene fluoride (PVDF), polytetrafluoroethylene (PTFE), and polypropylene (PP) have been used in OMD process. For the separation in the gas phase were also proposed new membrane materials such as magnetic mixed matrix membranes (Rybak et al. 2014, 2017). However, the achievement of strongly hydrophobic macroporous membranes is a condition of using the hybrid membranes in OMD process.

The driving force for the vapor transport in OMD is the partial pressure difference between the feed and brine, which is induced by a lower activity of water in the brine (Wang and Min 2011). Therefore, the OMD process can be carried out at room temperature and atmospheric pressure without a degradation of heat-sensitive components and losing some volatile components of liquid foodstuffs. This process is proposed for the removal of water from liquid food such as fruit and vegetable juices, milk, instant coffee and tea, and various non-food aqueous solutions, which are not thermally resistant (Ali et al. 2003; Bui and Nguyen 2005; Cissé et al. 2011). The inorganic salts (NaCl, CaCl_2_, MgCl_2_, and MgSO_4_) or organic solvents (glycerol and polyglycerol) can be used as stripping solutions. In the case of the concentration of liquid foodstuffs, the striping solution has to be non-toxic.

The water evaporation decreases while the vapor condensation increases the interfacial temperature in the layers adjacent to the membrane surfaces (temperature polarization) (Zambra et al. 2015), which causes a decrease of the driving force formed only by the concentration difference (osmotic distillation—OD). Moreover, a solute concentration near the membrane on the feed side is larger than that in the bulk, whereas the solute concentration on the extract side is lower than that in the bulk. This phenomenon is termed as the concentration polarization and it also leads to a driving force reduction (Ravindra Babu et al. 2008; Wang and Min 2011). Therefore, in the OMD variant, the feed is additionally heated, which increased its temperature to a similar or a little higher value than that of the brine, which increases the driving force and the higher process yields can be obtained compared to classical OD (Gryta 2013).

In the membrane processes, a permeation of water through a membrane causes the growth of concentration of the feed components and the deposits are often formed on the membrane surface (fouling and scaling), which is the subject of many works (Goh et al. 2013; Laqbaqbi et al. 2017). The scaling phenomenon on the permeate side (usually clean water) has not been considered so far. However, the saturated salt solutions flow on this side in the OMD process, which can cause the scaling. The formation of deposit hinders the mixing of condensed vapor with brine (brine dilution) and increases the resistance of heat transfer, which enhances a temperature of the interfacial layer: membrane (condensed vapor)/brine. These phenomena decrease a value of the mass driving force, and thus, they cause a decline of the permeate flux (Wang and Min 2011).

A basic problem, which impedes an industrial implementation of OMD, is a phenomenon of membrane wetting, which is often induced by scaling and membrane fouling (Laqbaqbi et al. 2017). The wettability of even a small fragment of the membrane wall causes the salts to penetrate from the stripping solution into the feed which deteriorates its quality and, as a result, can eliminate the possibility, e.g., juice concentration. Usually, the electrical conductivity of the juice is monitored to ensure the membrane integrity and hydrophobicity, and any salt leakage through the membrane was confirmed in several works (Bui and Nguyen 2005; Cissé et al. 2011; Vargas-Garcia et al. 2011; Zambra et al. 2015). However, these results were obtained during short periods of measurements (below 50 h); therefore, a significantly longer period (2.5 years) was used in this work for evaluation of membrane resistance for salt leakage. The presented studies were carried out using OMD installation with brine re-concentration through its natural evaporation, which allows to conduct OMD research lasting many weeks while maintaining a constant concentration of the stripping solution. It was studied whether a continuous contact of the saturated NaCl solution with the membranes does not cause the salt crystallization (scaling) and, as a consequence, the pore wetting.

## Experimental

An experimental installation used in the research was detailed described in the previous work (Gryta 2013) and presented in the Supplement. The installation was composed of the two closed cycles (feed and brine) connected to a capillary module installed in a vertical position. The module was equipped with 18 hydrophobic capillary polypropylene membranes (Accurel PP S6/2, Membrana GmbH—Germany) assembled inside the glass tube (diameter of 0.021 m and effective length of 0.83 m). The capillary membranes had the internal diameter of 1.8 mm and outer diameter of 2.6 mm, and the porosity was 73% (the manufacturer’s data). The membranes were positioned in every second mesh of six sieve baffles, arranged across the module housing within a distance of 0.15 m. The feed flow inside the capillaries and the total membrane area calculated for the lumen side amounted to 0.084 m^2^.

The saturated NaCl (technical grade, ChemPur, Poland) solutions were used as a stripping solution (about 317 g NaCl/L). The brine (6 L) was storage inside a rectangular open tank. A pyramid tower composed of 17 Białecki rings (80 mm in diameter and high) manufactured by DOLSIN (Poland) was placed inside this tank (over the brine surface). The NaCl solution after leaving the module was directed towards the top of the tower where it was sprayed on the surface of the first top ring. The evaporation surface created by the Białecki rings (0.7 m^2^) was found to be large enough to maintain a constant concentration of brine through the natural evaporation.

The membrane wetting and polypropylene resistance on the saturated NaCl solution were mainly studied in this work. Therefore, distilled water was used as a feed, which allowed to limit the influence of polarization phenomenon on this side of the membrane. The brine and feed temperatures at the module inlets were in the range of 295–307 K. Similarly to the industrial conditions the feed, brine and air surrounding the OMD installation were not thermostated during the studies. The permeate flux (water vapor) transferred through the membrane was calculated on the basis of changes in water volume in the feed tank (5 L). This tank was refilled with distilled water usually once a day, adding 0.8–1.5 L of water. Assuming that this volume was determined with accuracy up to 5 mL, an error of determination of permeate flux amounted to less than 1%.

The feed and brine were pumped using magnetic centrifugal pumps (TE-3-MD-HC) manufactured by Little Giant Pump (USA). The flow rates of feed and brine were 1.3 ± 0.1 and 0.25 ± 0.03 m/s, respectively. The hydrostatic pressure (module inlets) of both circulating feed and brine was measured by a pressure gauge (34–35 kPa feed and 24–36 kPa brine). The stream temperatures were measured using electronic thermometers with ±0.1 K accuracy.

The solute concentration expressed as the total dissolved solids (TDS–NaCl mode) and the electrical conductivity of water were measured with 6P Ultrameter (Myron L Company, USA). Before the measurements, the brine samples were 10 times diluted with distilled water. An air temperature and the relative humidity were measured by electronic hygrometer AZ8829 (AZ-Instruments, Poland) connected with computer software TRLOG v.3.4.

The membrane and deposit morphology was studied using a Hitachi SU70 Field Emission Scanning Electron Microscope (FESEM) with Energy-dispersive X-ray Spectrometer (EDS) purchased from Thermal Scientific. The applied software of EDS detector enables to make the analysis of elements with low molecular weight (from Li). The samples for cross-sectional observations were prepared by fracture of the capillary membranes in liquid nitrogen. All the samples were sputter coated with gold and palladium.

The measurements of phase transition temperatures of polymers can be useful indicator for evaluation of the extent of polymer degradation. The thermal properties of used polypropylene membranes were evaluated by differential scanning calorimetry (DSC). The measurements were performed with the use of Q1000 TA Instruments apparatus, at heating–cooling rate of 10 deg/min.

## Results and discussion

### The influence process parameters on the permeate flux

**Fig. 1:**
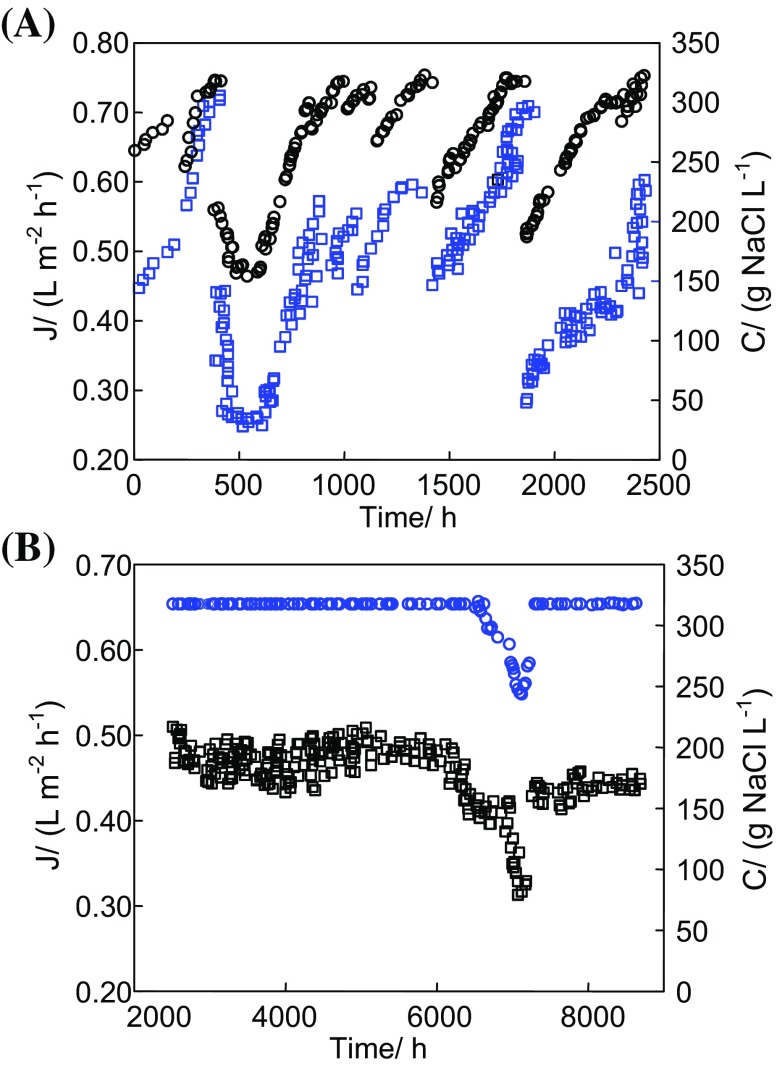
Changes of the permeate flux (J—*open square*) and brine concentration (C—*open circle*) during OMD module exploitation

The studies of OMD process were realized (with few longer breaks) over a period of 2.5 years, obtaining almost 9000 h of operation of the membrane module (Fig. 1).

In the case when a break in the OMD studies was lasting over 2 weeks, the brine cycle was rinsed and filled with distilled water. As a result, the applied Accurel PP S6/2 membranes were in contact with NaCl solution and water over the entire period of the investigations. The results presented in Fig. 1 confirmed that the used membrane module maintained a good efficiency during the studies. The obtained changes of value of the permeate flux resulted from variations of particular process parameters, such as the concentration of brine and the feed temperature. The influence of these parameters on the course of process was presented in the consecutive figures.

**Fig. 2:**
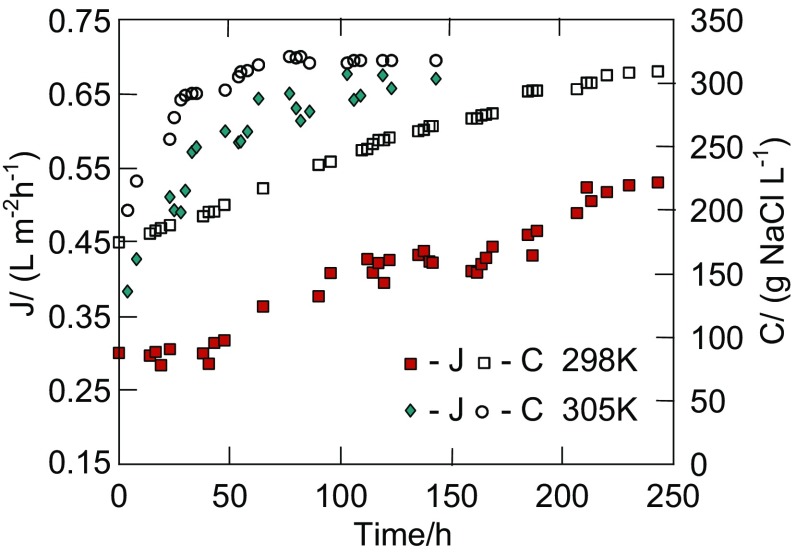
The OMD process with a continuous regeneration of stripping solution. In the case of *T*_F_ = 305 K, the feed was additionally heated

During the first 2400 h of OMD studies (Fig. 1a), the efficiency of evaporation system (used for brine re-concentration) for different process parameters was tested. Independently of the process conditions, the concentration of brine was stabilized at 310–317 g NaCl/L (Fig. 2). The brine and feed in the OMD installation were heated by heat released as a part of energy used for pumping the solutions (centrifugal pumps), which caused the process temperature in the installation to stabilize at 298 K. The process temperature increased to 305 K when the additional heating of feed was switched on. This slight increase of temperature significantly accelerated the water evaporation from brine, and the time of its concentration from 170 to 317 g NaCl/dm^3^ was shorted from 250 to 60 h (Fig. 2). In this case, the permeate flux was increased over two-fold.

**Fig. 3:**
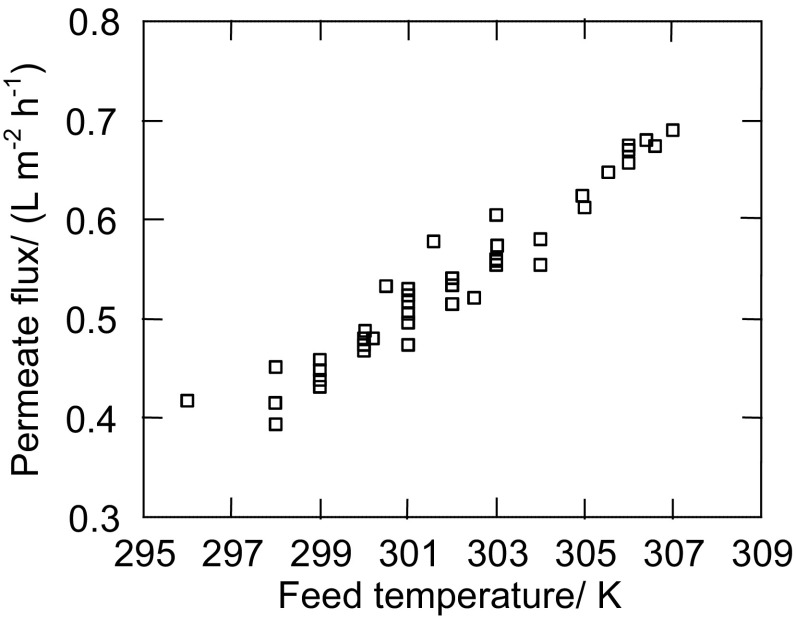
The influence of feed temperature on the permeate flux during OMD process

The external and internal surfaces of the assembled 17 Białecki rings created the evaporation surface equal to 0.7 m^2^, which was found to be large enough to maintain a brine concentration close to the saturation state during the OMD studies (Fig. 1b, e.g., 2500–6000 h). In the case of saturated brine, an elevation of feed temperature from 297 to 307 K caused the increase of the permeate flux from 0.4 to 0.7 L/m^2^h (Fig. 3). Despite a significant increase of the amount of water (vapor) transferred from the feed to brine, the brine concentration was practically not changed. This resulted from the fact that the amount of water evaporated from surface of Białecki rings was larger than the permeate flux, a visible result of which was the salt crystallization in the brine tank and on the surface of Białecki rings. A reduction of the amount of Białecki rings by a half limited the evaporation of water from brine, which caused a decline of the brine concentration and, as a result, a decrease of the permeate flux (Fig. 1b, 6400–7300 h). In the tested OMD installation, a saturated NaCl solution was achieved when the evaporation tower was composed from at least 14 Białecki rings (165 rings per 1 m^2^ of membranes). The re-concentration of brine proceeded efficiently for such a number of rings, irrespective of humidity of air surrounding the OMD installation (Gryta 2013).

**Fig. 4:**
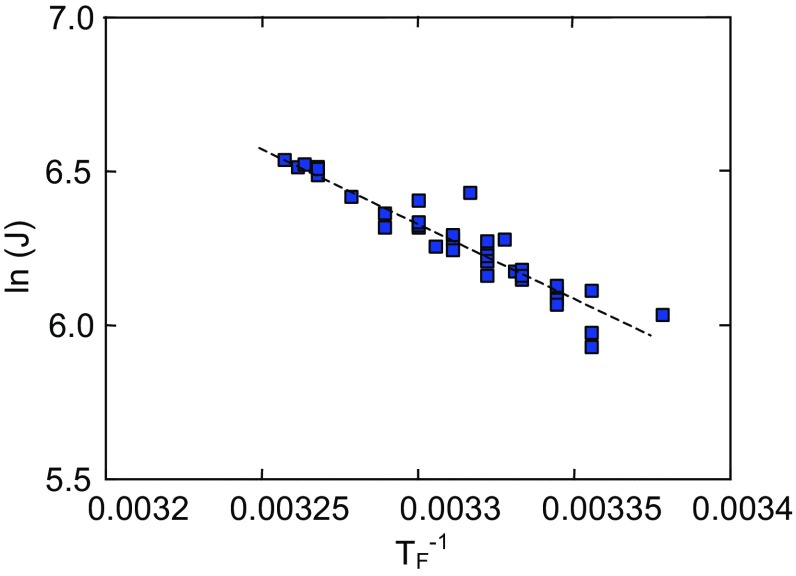
Relation of ln(permeate flux—J) versus 1/*T*_F_. Feed temperature in range of 298–303 K

The influence of feed temperature on the OMD thermal efficiency can be evaluated based on the Arrhenius-type equation (Godino et al. 2009; Kujawa et al. 2015):

1 $$J = \exp \left( { - E/RT} \right),$$

where *R* is the gas constant and *E* is the apparent activation energy associated with the flux. In the above equation, the apparent energy expressed not only the evaporation energy but also includes both the losses of the heat and heat which is transferred from the condensation layer to the evaporation surface. From the slope of the trend line in Fig. 4, representing the natural logarithm of water fluxes against a reciprocal of the absolute temperature of the feed, the estimated activation energy was equal to 41 kJ/mol. This value is close to the theoretical value (43 kJ/mol), which confirmed that similar feed and brine temperatures considerably reduce the heat losses in OMD process. The larger values of the apparent activation energy (51 kJ/mol) was estimated, when a significantly larger temperature difference across the membrane was applied (Kujawa and Kujawski 2015).

**Fig. 5:**
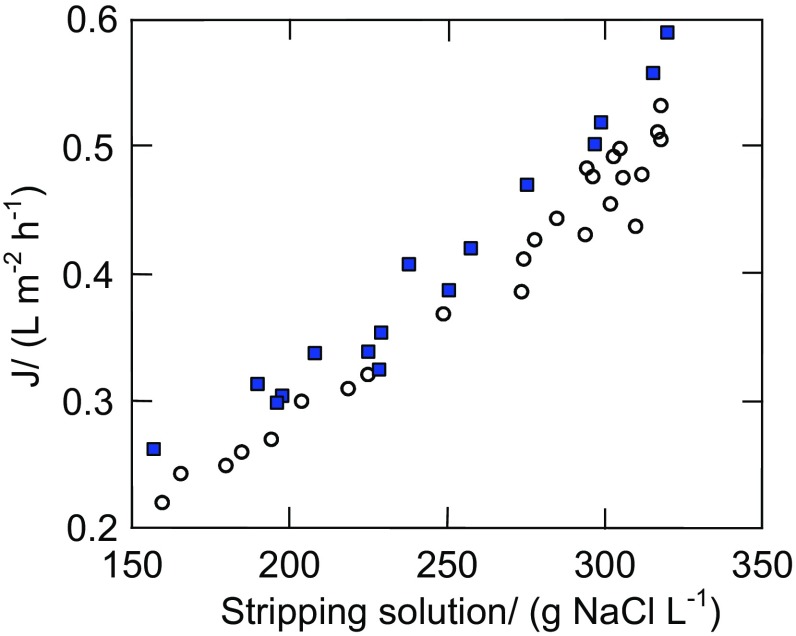
The influence of stripping solution concentration on the permeate flux (J). Feed temperature:* open circle*—301 K,* filled square*—306 K

The results presented in Fig. 5 confirmed that a maintenance of the brine concentration close to the saturation state is important to achieve a high yield of the OMD process (Cassano et al. 2004; Wang and Min 2011; Zambra et al. 2015). In the case under study, a reduction of brine concentration from 320 to 300 g NaCl/L caused a decrease of the permeate flux from 0.6 to 0.5 L/m^2^h. A further decline of yield by a similar value (from 0.5 to 0.4 L/m^2^h) was obtained when the brine concentration was reduced by a larger value, i.e., from 300 to 240 g NaCl/L (Fig. 5). This decrease of the influence of brine concentration allows to obtain an average level of process yield using a periodical re-concentration of brine in the evaporator (Cissé et al. 2011). However, with regard to the relative low yields of the OMD process, it is recommended to maintains the maximum permeate flux, which requires a continuous regeneration of extracting solution (Ali et al. 2003).

**Fig. 6:**
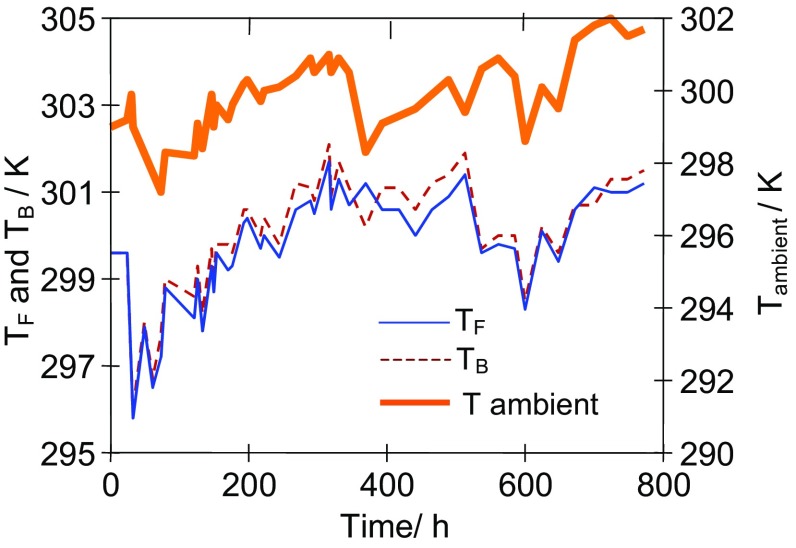
The effect of ambient temperature on the changes of feed (*T*_F_) and stripping solution (*T*_B_) temperatures

The results presented in Fig. 1b (e.g., 3000–5000 h) indicate that the permeate flux was varied during the process, despite the fact that the brine concentration was constant (saturated solution). The OMD installation was not thermostated, and a reason of observed flux variations was associated with a fluctuation of the feed and brine temperatures, which resulted from the changes of temperature of air surrounding the OMD installation (Fig. 6). A difference in the partial vapor pressure (driving force) caused by the concentration difference is not too large; hence, even a small change of solutions temperature substantially affected the OMD yield (Fig. 3). In the laboratory installations, the feed and brine temperature is usually stabilized at the same level by the heat exchange systems (Warczok et al. 2007, Zambra et al. 2015). However, such a solution would be too expensive in the industrial OMD installations, where only a cooling of brine is usually carried out, the temperature of which significantly increases due to the permeate condensation (Ali et al. 2003; Cissé et al. 2011). In our case, a cooling of brine (by about 0.2–0.4 K) took place due to water evaporation from the surface of Białecki rings. As a result, the brine temperature at the inlet of OMD module was close to or lowers by about 0.1–0.2 K than the inlet feed temperature. This is an advantage of the tested OMD system because the driving force for vapor transport is increased.

### Membrane wettability

During a long-term exploitation of the hydrophobic membranes, the amount of pores that were filled by separated solutions (membrane wetting) is gradually increased (Goh et al. 2013). As a result, a systematic decline of the permeate flux is observed. Moreover, when the pores are wetted through the total thickness of the wall, a brine leakage through the wetted pores was also increased. In the case of membrane distillation, a feed leakage caused a systematic increase of the distillate conductivity (Gryta 2017). In the OMD process, the brine flows on the distillate side; thus, it should be expected that a salt would penetrate into the feed. In order to accurately examine the intensity of this phenomenon, the distillate water was used as a feed in the presented studies, which also allowed to determine the changes of the maximal permeate flux during a long-term operation of the OMD installation.

**Fig. 7:**
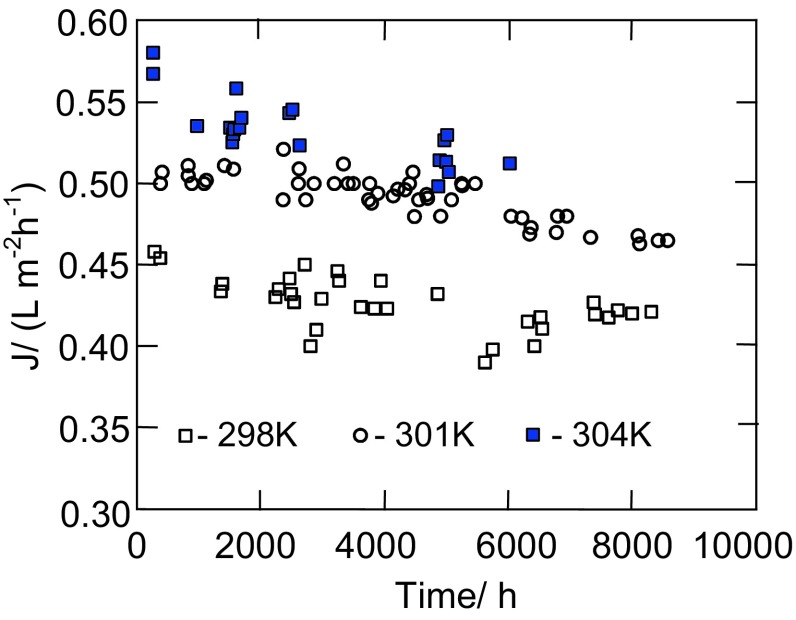
Changes of the permeate flux during OMD process at different feed temperatures

A fluctuation of the permeate flux (Fig. 1b) caused by the changes of process temperature hinders the above analysis; therefore, the selected results of studies obtained for similar conditions of the OMD process are presented in Fig. 7. The obtained results indicate that the maximal permeate flux was reduced by 10–20% over a studied period. Taking into consideration that the module was tested for 2.5 years, it should be recognized that a decrease of the yield was small, which confirms a good suitability of the polypropylene membranes for OMD process, which was also reported in other works (Ali et al. 2003; Bui and Nguyen 2005; Cassano et al. 2004; Zambra et al. 2015).

**Fig. 8:**
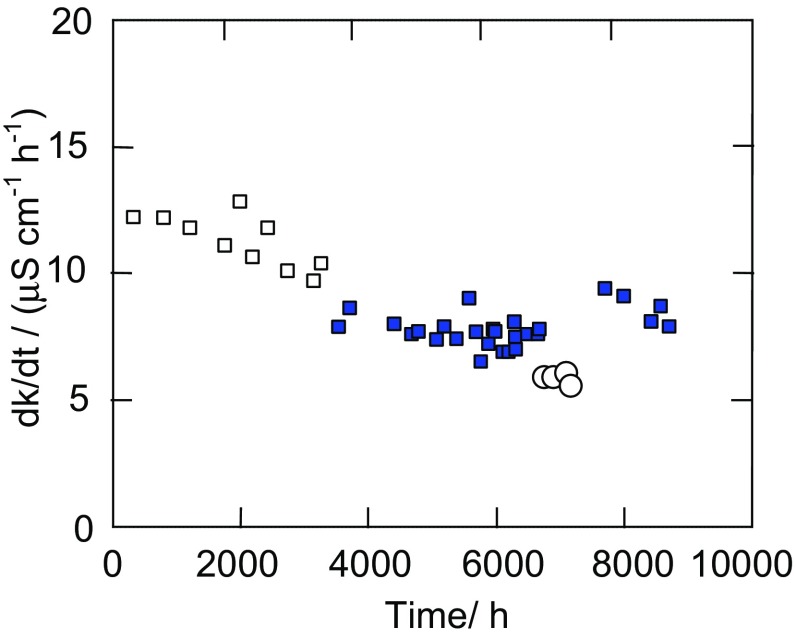
Changes of the electrical conductivity (k) of water (on the feed side) during OMD process. Pressure on the brine side:* open square*—36 kPa,* filled square*—34 kPa,* open circle*—24 kPa

The studies of the electrical conductivity indicated that a small leakage of brine into the feed occurred (Fig. 8). However, the salt concentration in the feed was very low, and due to the fact that the brine was a saturated solution, the salt rejection was close to 100% in all cases.

It is worthy to notice that the measured growths of conductivity value did not build up during the consecutive weeks of process duration. It was found that the changes presented in Fig. 8 were only depended on a value of hydrostatic pressure. During the initial period of 3000 h, this pressure amounted to 36 kPa on the brine side, and that of the feed was 34 kPa, which facilitates a leakage of brine to the feed. A decrease of pressure on the brine side to 34 kPa caused the conductivity growths to reduce from 12 to 7–8 μS/cm per 1 h. A consecutive reduction of pressure to 24 kPa on the brine side caused a further decrease of conductivity growth to 6 μS/cm during 1 h of OMD (Fig. 8, points marked by circle). Such results indicated that the membranes were wetted in a slight degree, probably mainly during the first 50–100 h of module exploitation (Gryta 2017). Thus, the main reason of the observed systematic decline of the process yield (Fig. 7) was the formation of deposits on the membrane surface, which was discussed in the subsequent section. Moreover, after a disconnection of module from OMD installation, a formation of NaCl crystals was observed at the end of one of the module head. Therefore, a reason of observed leakage (Fig. 8) could be associated with a damage of connection of the module head with glass tube.

### Membrane scaling

After completing the OMD studies, the membranes taken out from the OMD module were still flexible, which testified that polypropylene did not undergo the significant degradation over examined period. The membrane samples collected for SEM examinations were taken at a distance of 5 cm from the inlet and 5 cm from the outlet of module.

Polypropylene is a semicrystallite polymer and the changes of the crystallinity degree (X) affected its mechanical properties. The changes in the polymer matrix were studied using the DSC analysis (Table 1). The new polypropylene membranes were characterized by a value of the crystallinity degree at a level of 40%, and only a small increase to a value of 40.6% was detected for the membranes collected from OMD module. Moreover, the values of other parameters, such as melting (*T*_m_) and crystallization (*T*_C_) temperatures, and melting (∆*H*_m_) and crystallization (∆*H*_C_) enthalpy were similar, which confirmed a low level of PP membrane degradation despite 2.5 years of MD module exploitation.

**Table 1:** Results of DSC analysis

Sample	*T*_m_ (°C)	∆*H*_m_ (J/g)	*T*_C_ (°C)	∆*H*_C_ (J/g)	*X* (%)
New membrane	162.5	84	115.1	92	40.6
After OMD	162	83	118.5	90	40

**Fig. 9:**
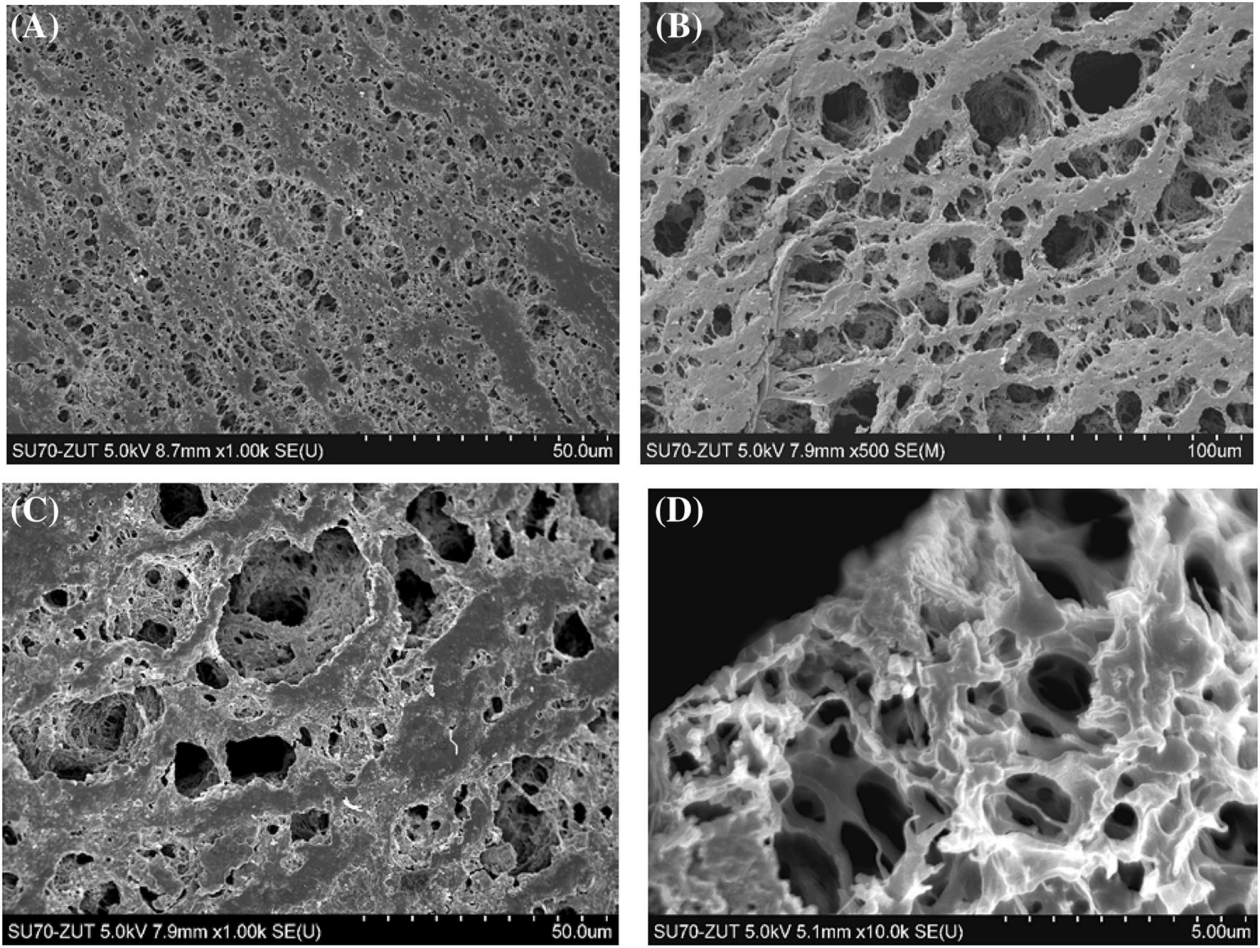
SEM images of the surface of membrane collected at the module inlet. **a** Internal surface, **b** external surface—on the brine side, **c** external surface with deposit, **d** cross section

**Fig. 10:**
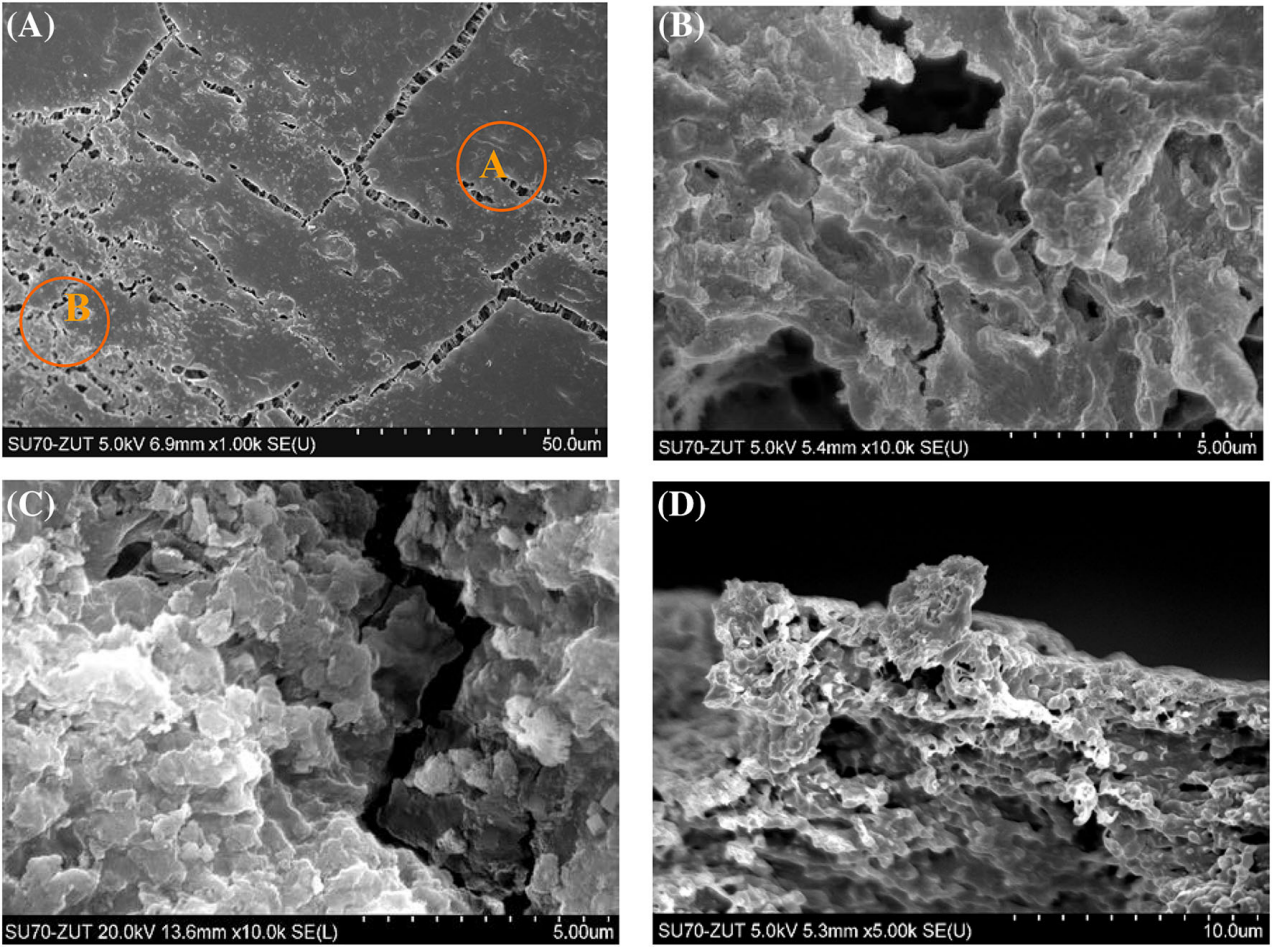
SEM images of the surface of membrane collected at the module outlet. **a** Membrane covered by deposit, **b** “A” area magnified, **c** “B” area magnified, **d** membrane cross section

The performed SEM observations confirmed that numerous deposits were formed on the membrane surface on the brine side (Figs. 9, 10). A smaller amount of deposits was formed in the vicinity of the module inlet. Although the salt crystallization proceeded in the brine tank due to oversaturation caused by brine evaporation, the presence of NaCl crystallites was not found on the membrane surface. In the OMD modules, the condensation of permeate decreases the salt concentration on the surface layer on the brine side, which facilitates to limit the salt scaling.

**Fig. 11:**
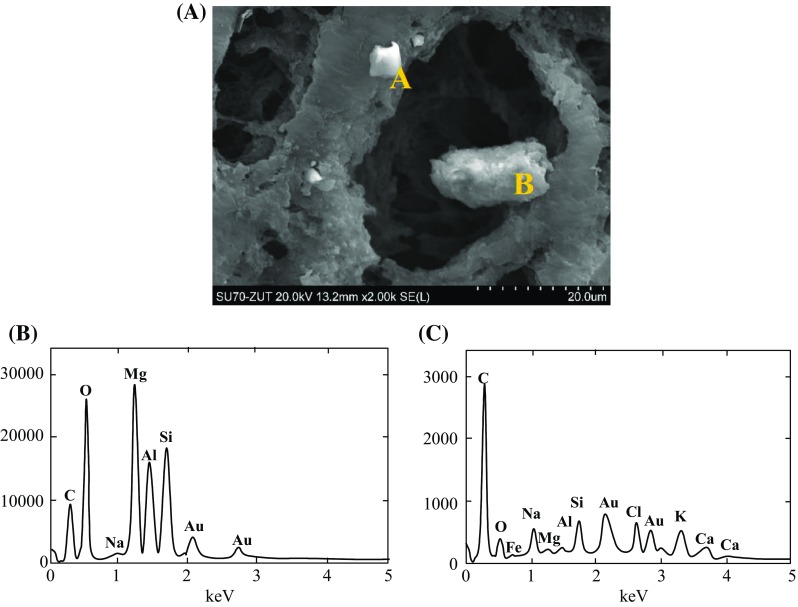
Results of SEM–EDS analysis of membrane sample collected at the module inlet. **b**, **c**—composition of A and B crystals shown in **a**

In agreement with the expectations, the internal membrane surface (on the feed side) was clean (Fig. 9a), and its image was similar to that obtained for new membranes. This indicates that 2.5-year contact of the membranes with distilled water did not cause the significant changes in their morphology, which confirms the results of previous studies (Gryta 2005). The external surface of membrane sample collected near the module inlet was only partly covered by the deposit. The pore structure characteristic for new membranes was observed on the parts of surface without deposit (Fig. 9b). On the other parts of membrane surface, the formed amorphous deposit partly covered the pore inlets (Fig. 9c). The examination of cross sections revealed that this deposit was very thin (0.5–2 μm) and did not penetrate into the pore interior (Fig. 9d). The SEM–EDS examination revealed that this deposit mainly contained silicone and oxygen. Few crystallites also occurred on the membrane surface, some of these crystallites contained mainly O and Si, and smaller amounts of Mg, Al, Na, Fe, K, Ca, and Cl (Fig. 11).

**Fig. 12:**
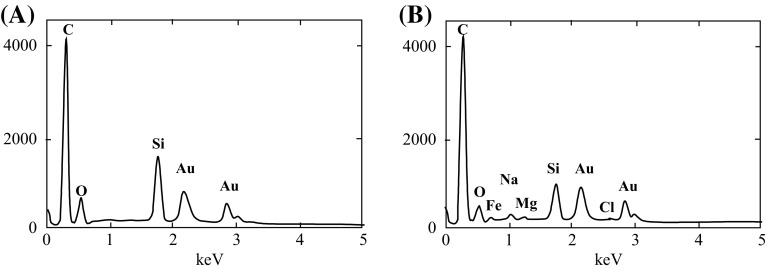
Results of SEM–EDS analysis of deposits formed on the membrane surface collected at the module outlet. **a** Deposit shown in Fig. 10b, **b** deposit shown in Fig. 10c

The SEM observations demonstrated that the membranes near the outlet of the module were covered by a more compact layer of deposits (Fig. 10). The SEM–EDS examinations revealed (Fig. 12) that the deposit mainly contained Si and O, and smaller amounts of Na, Mg, and Fe. Thus, it can be assumed that the hydrated sodium silicate was mainly deposited on the membrane surface. With regard to this, the majority of fractures visible in the deposit in Fig. 10a was formed during the preparation of sample for SEM examinations (natural drying). However, the observations performed under larger magnifications demonstrated that the porous fragments were also present in the deposit (Fig. 10b, c). The SEM observations of membrane cross sections (Fig. 10d) revealed that the formed deposit was considerably thicker (5–10 μm) than that observed at the module inlet. The deposit also partially fulfilled the surface pores, particularly those with large diameters (e.g., 10–20 μm). However, it was found that the formed deposit did not generally exhibit a strong tendency to penetrate into the interior of the membrane wall.

## Conclusions

The long-term studies confirmed the suitability of polypropylene membrane Accurel PP S6/2 for the realization of OMD process. The membrane did not undergo the wettability and preserved its flexibility during the contact with water and saturated NaCl solution. This testified that the degradation of polypropylene was negligible during a studied period of 2.5 years. The results of DSC analysis confirmed a low level of PP membrane degradation.

The performed studies confirmed that re-concentration of salt solutions using the natural evaporation can be an efficient method of maintaining a constant concentration of the stripping solution in OMD process.

The feeding of OMD module with saturated NaCl solution (stripping solution) did not caused the salt crystallization on the membrane surface.

It can be expected that the contaminations (e.g., silicon compounds) presented in the salt used for the preparation of stripping solution may form the deposits on the membrane surface. In the case under study, a thin layer of amorphous deposit was formed on the membrane surface, mainly containing O and Si. The presence of such deposit caused the permeate flux to decrease by 10–20% in the OMD process over studied period of 2.5 years.

## Electronic supplementary material

Below is the link to the electronic supplementary material.

Supplementary material 1 (DOC 19209 kb)
