# Supplementary material for: The long-term studies of osmotic membrane distillation
Source: Chem Zvesti. 2017 Aug 3;72(1):99–107. doi: 10.1007/s11696-017-0261-1 (PMC5760615; doi:10.1007/s11696-017-0261-1)
Supplement: Supplementary file 1 — Supplementary material 1 (DOC 19209 kb) [file 11696_2017_261_MOESM1_ESM.doc]

Supplement

The long-term studies of osmotic membranes distillation

**Marek Gryta**

Experimental set-up

The experimental set-up used in the studies is schematically presented in Figs 1A and 2A. The flux of permeate obtained during the OMD process was calculated on the basis of changes in the water volume in the feed tank. For this purpose, after starting the pilot plant and achieving the constant parameters of its operation the feed tank was filled-up with water and the feed level was read from a pipette scale (8 - Fig. 1A and Fig.3B). During the OMD process the water evaporated through the membranes, and the feed loss was refilled usually once a day in the feed tank, which also enables the determination of the average permeate flux over the period of experiment duration.

Fig. 1A. The OMD pilot plant. 1- OMD module, 2 – manometer, 3 – brine tank, 4 – pump, 5 – heat exchanger, 6 – thermometer, 7 – feed tank, 8 – pipette, 9 – filter, 10- - Białecki rings, 11- net


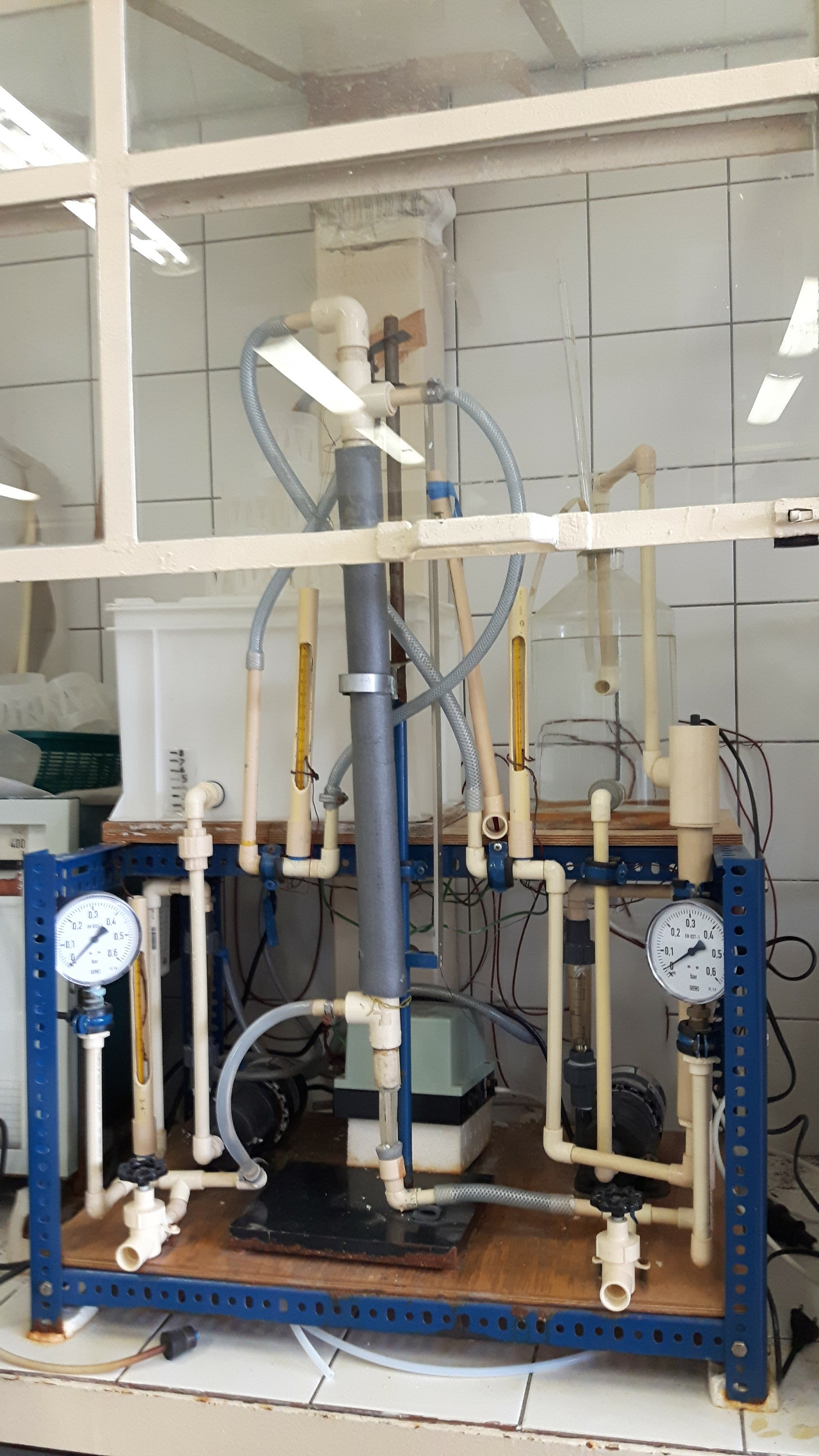


Fig.2A. General view of the OMD installation


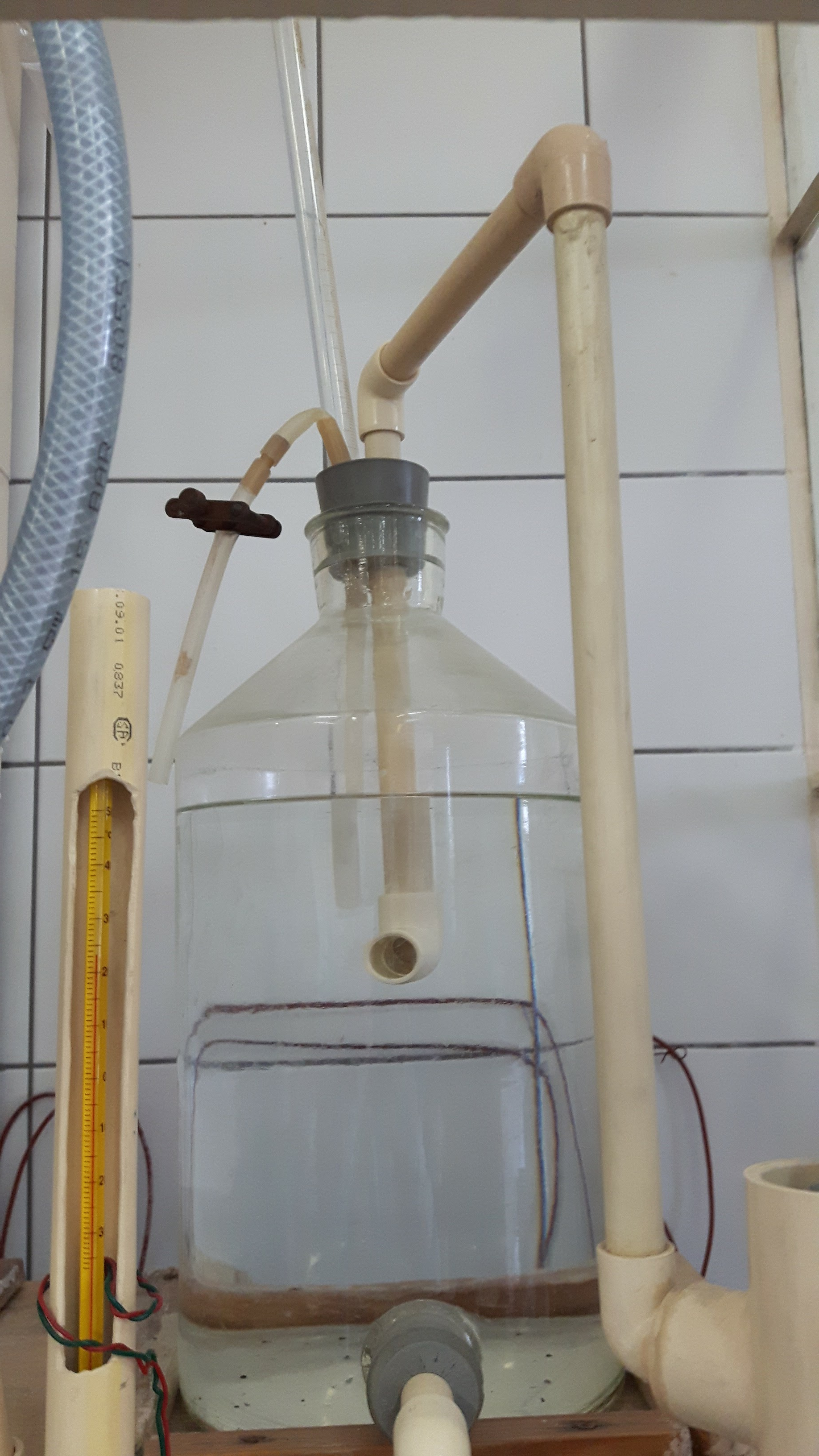


Fig.3B. Feed tank with water system dosing


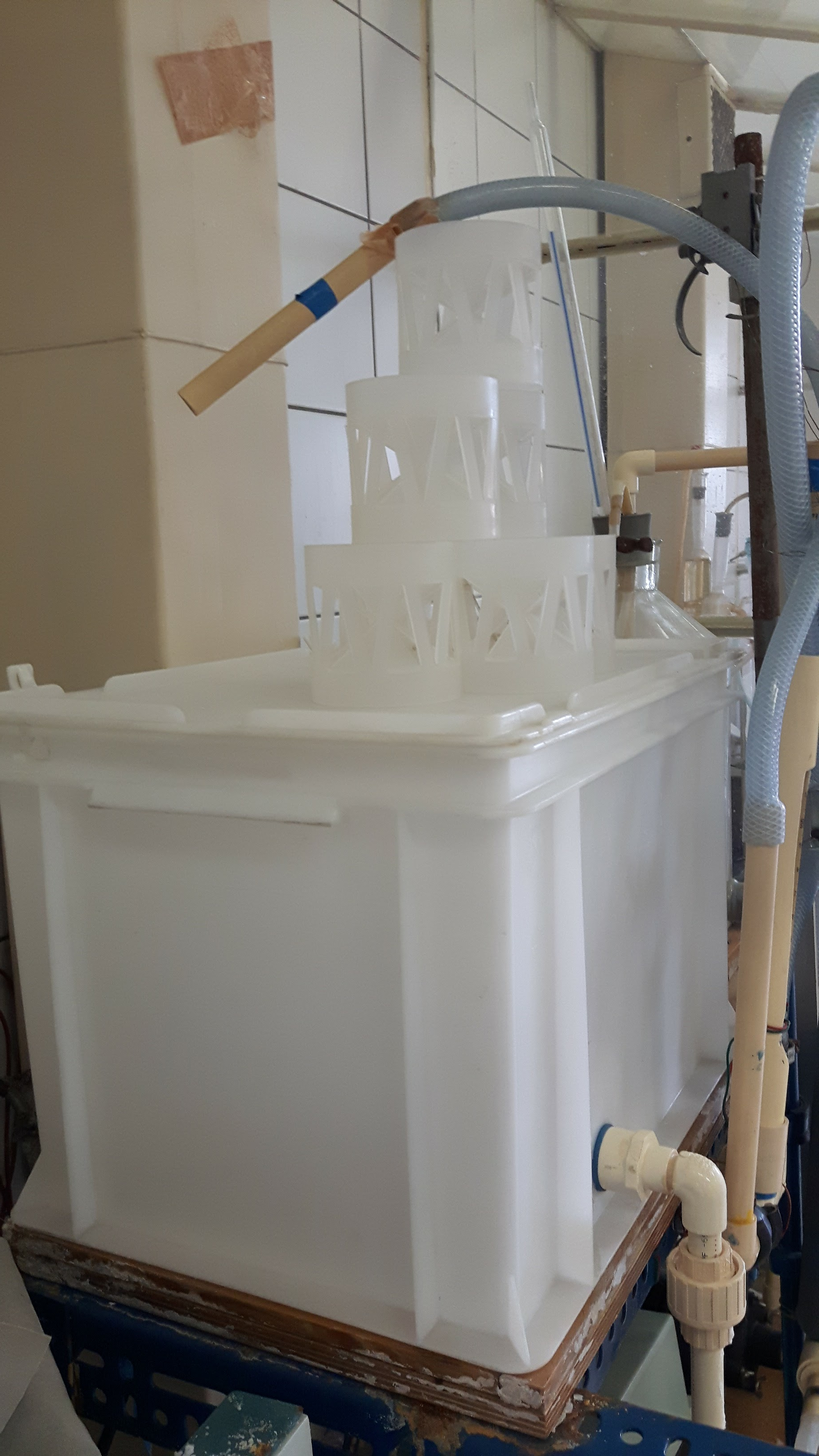


Fig. 4A. Brine tank with Białecki rings

Fig. 5A. The images of used Białecki ring (A), and B) rings covered by NaCl deposit
